# Application of the Moran Model in Estimating Selection Coefficient of Mutated CSF3R Clones in the Evolution of Severe Congenital Neutropenia to Myeloid Neoplasia

**DOI:** 10.3389/fphys.2020.00806

**Published:** 2020-09-17

**Authors:** Khanh N. Dinh, Seth J. Corey, Marek Kimmel

**Affiliations:** ^1^Irving Institute for Cancer Dynamics and Department of Statistics, Columbia University, New York, NY, United States; ^2^Departments of Pediatric and Cancer Biology, Cleveland Clinic, Cleveland, OH, United States; ^3^Departments of Statistics and Bioengineering, Rice University, Houston, TX, United States; ^4^Department of Systems Biology and Engineering, Gliwice, Poland

**Keywords:** clinical data, G-CSF receptor (G-CSFR), recurrent mutation, myeloid neoplasia, Moran model, selective advantage

## Abstract

Bone marrow failure (BMF) syndromes, such as severe congenital neutropenia (SCN) are leukemia predisposition syndromes. We focus here on the transition from SCN to pre-leukemic myelodysplastic syndrome (MDS). Stochastic mathematical models have been conceived that attempt to explain the transition of SCN to MDS, in the most parsimonious way, using extensions of standard processes of population genetics and population dynamics, such as the branching and the Moran processes. We previously presented a hypothesis of the SCN to MDS transition, which involves directional selection and recurrent mutation, to explain the distribution of ages at onset of MDS or AML. Based on experimental and clinical data and a model of human hematopoiesis, a range of probable values of the selection coefficient *s* and mutation rate μ have been determined. These estimates lead to predictions of the age at onset of MDS or AML, which are consistent with the clinical data. In the current paper, based on data extracted from published literature, we seek to provide an independent validation of these estimates. We proceed with two purposes in mind: (i) to determine the ballpark estimates of the selection coefficients and verify their consistency with those previously obtained and (ii) to provide possible insight into the role of recurrent mutations of the G-CSF receptor in the SCN to MDS transition.

## 1. Introduction

Bone marrow failure (BMF) syndromes, such as severe congenital neutropenia (SCN) are leukemia predisposition syndromes. In addition to SCN, these heterogeneous groups of disorders include Fanconi anemia, dyskeratosis congenita, Diamond-Blackfan anemia, Shwachman-Diamond syndrome, and *GATA2* deficiency (West and Churpek, 2017; Kennedy and Shimamura, 2019). Each of these clinically defined disorders are monogenic with mutations in one or more genes in a pathway. For example, Fanconi anemia results from germline mutations in genes involved in DNA repair and Diamond-Blackfan anemia in ribosome structure (Oyarbide et al., 2019). What is less well-understood are the somatic mutations that arise during transformation of a BMF syndrome to myeloid neoplasia (Rafei and DiNardo, 2019).

Focusing on the SCN to MDS to AML transition, individuals with a germline mutation in the *ELANE* gene develop at early age a severe neutropenia (Touw, 2015). This profound neutropenia makes them susceptible to recurrent infections, which can be only partly managed by antibiotics. Treatment introduced in the 1990s involves administration of large doses of recombinant human granulocyte colony stimulating factor (G-CSF), which boosts neutrophil production (Bonilla et al., 1989). Unfortunately, in about 30% of patients, either myelodysplatic syndrome (MDS), a preleukemic disorder, or acute myeloid leukemia (AML) emerges. In 70% MDS or AML cases arising from SCN, somatic mutations of the G-CSF Receptor (*CSF3R*) occur (Link, 2019). These are almost always nonsense mutations. The truncated *CSF3R* affects altered signaling, gene expression, and phenotype within the neutrophil lineage. There is enhanced proliferation and impaired neutrophilic differentiation to G-CSF.

Stochastic mathematical models have been conceived, which attempt to explain the transition of SCN to MDS and then to AML, in the most parsimonious way, using suitable extensions of standard processes of population genetics and population dynamics, such as the branching (Kimmel and Corey, 2013) and the Moran processes (Wojdyla et al., 2019). Specifically, the latter paper presented a hypothesis of the SCN → MDS transition, which involves the Moran process with directional selection (Durrett, 2008) and recurrent mutation, to explain the distribution of ages at onset of MDS or AML. As argued in Wojdyla et al. (2019), starting in the fetal life, *CSF3R* mutations arise as a random process and are selected for when G-CSF is administered to boost neutrophil production. Based on experimental and clinical data and a model of human hematopoiesis, a range of probable values of the selection coefficient *s* and mutation rate μ have been determined. These estimates lead to predictions of the age at onset of MDS or AML, which are consistent with the clinical data.

In the current paper, based on data extracted from published literature, we seek to provide an independent validation of these estimates. We will use the model of evolution of the mutant receptors in the hematopoietic stem cells (HSC) in the bone marrow in the form of a Moran process with selection and recurrent mutation. This is the same process we used in Wojdyla et al. (2019), except that here, to simplify computations, we assume constant HSC population size and develop an analytical approximation of the expected values of the mutant receptor occurrence among HSC under the assumption that initial count of mutants is already substantial (Methods and Data). We proceed with two purposes in mind. Our first purpose is to determine the ballpark estimates of the selection coefficients and verify their consistency with those obtained in Wojdyla et al. (2019). Our second purpose is to provide insight into the relative role of recurrent mutations of the G-CSF receptor in the SCN to MDS transition.

## 2. Methods and Data

### 2.1. Moran Process

In the monograph by Durrett (2008), the Moran process with selection is defined as follows

- Constant population of *N* individuals.
- At each discrete time moment, a randomly chosen individual dies, and, at the same moment, another randomly chosen individual proliferates (for mathematical completeness, it can be the same individual).
- In the model with directional selection, there are individuals of two types: wildtype (WT) and mutant (M) and the choice of individual that proliferates is biased. The wildtype is chosen with weight 1 − *s*, *s* ∈ (0, 1).

It is instructive to consider the discrete-time case first. Let us denote the number of mutants by *i*. There are four possibilities

- WT dies, with probability (*N* − *i*)/*N*
  - WT proliferates, with probability (1 − *s*)(*N* − *i*)/[(1 − *s*)(*N* − *i*) + *i*]
  - M proliferates, with probability *i*/[(1 − *s*)(*N* − *i*) + *i*]
- M dies, with probability *i*/*N*
  - WT proliferates, with probability (1 − *s*)(*N* − *i*)/[(1 − *s*)(*N* − *i*) + *i*]
  - M proliferates, with probability *i*/[(1 − *s*)(*N* − *i*) + *i*]

Only the WT → M and M → WT options lead to change in the number of mutants

$$p_{i,i+1}=\frac{N-i}{N}\frac{i}{\left[(1-s)(N-i)+i\right]},$$

$$p_{i,i-1}=\frac{i}{N}\frac{\left(1-s)(N-i\right)}{\left[(1-s)(N-i)+i\right]},$$

the M → M and WT → WT options jointly contribute to *p*_*i, i*_. States {0} and {*N*} are absorbing. The probability of being eventually absorbed in {*N*}, if at time 0 there are *i* mutants, is equal to

$$P\left[T_{N}<T_{0}\right]=\frac{1-{\left(1-s\right)}^{i}}{1-{\left(1-s\right)}^{N}}$$

in the case with selection, which leads to

$$P\left[T_{N}<T_{0}\right]=i/N$$

in the neutral case.

The continuous-time version is defined by transition intensities

$$q_{i,i+1}=(N-i)\frac{i}{N},q_{i,i-1}=i\frac{\left(1-s)(N-i\right)}{N},$$

which have different denominators than the transition probabilities. However, they lead to the same absorption formula. The expected time to absorption in {*N*} (fixation of the mutant) has a commonly used asymptotics

$$E_{1}\left(T_{N}\right)\sim \frac{2}{s}\ln\left(N\right)$$

as *N* → ∞ in the case with selection, which however is not very accurate.

### 2.2. Time-Continuous Moran Process With Directional Selection and Recurrent Mutations

A time-continuous Moran process with directional selection may be supplemented with recurrent mutation by adding a term of the form μ(*N* − *i*) to the *q*_*i,i*+1_ transition intensity. This can be interpreted as an equal and independent chance μ*Δt* + *o*(Δ*t*), for each of the *N* − *i* WT cells, of becoming a mutant in a short time interval (*t,t*+Δ*t*). The complete set of transition rules for the chain {*X*(*t*), *t* ≥ 0} assumes the form

(1) $$q_{i,i+1}=\left(N-i\right)\frac{i}{N}+\mu \left(N-i\right),\;q_{i,i-1}=i\frac{\left(1-s\right)\left(N-i\right)}{N},i=0,\ldots N.$$

**Figure 1:**
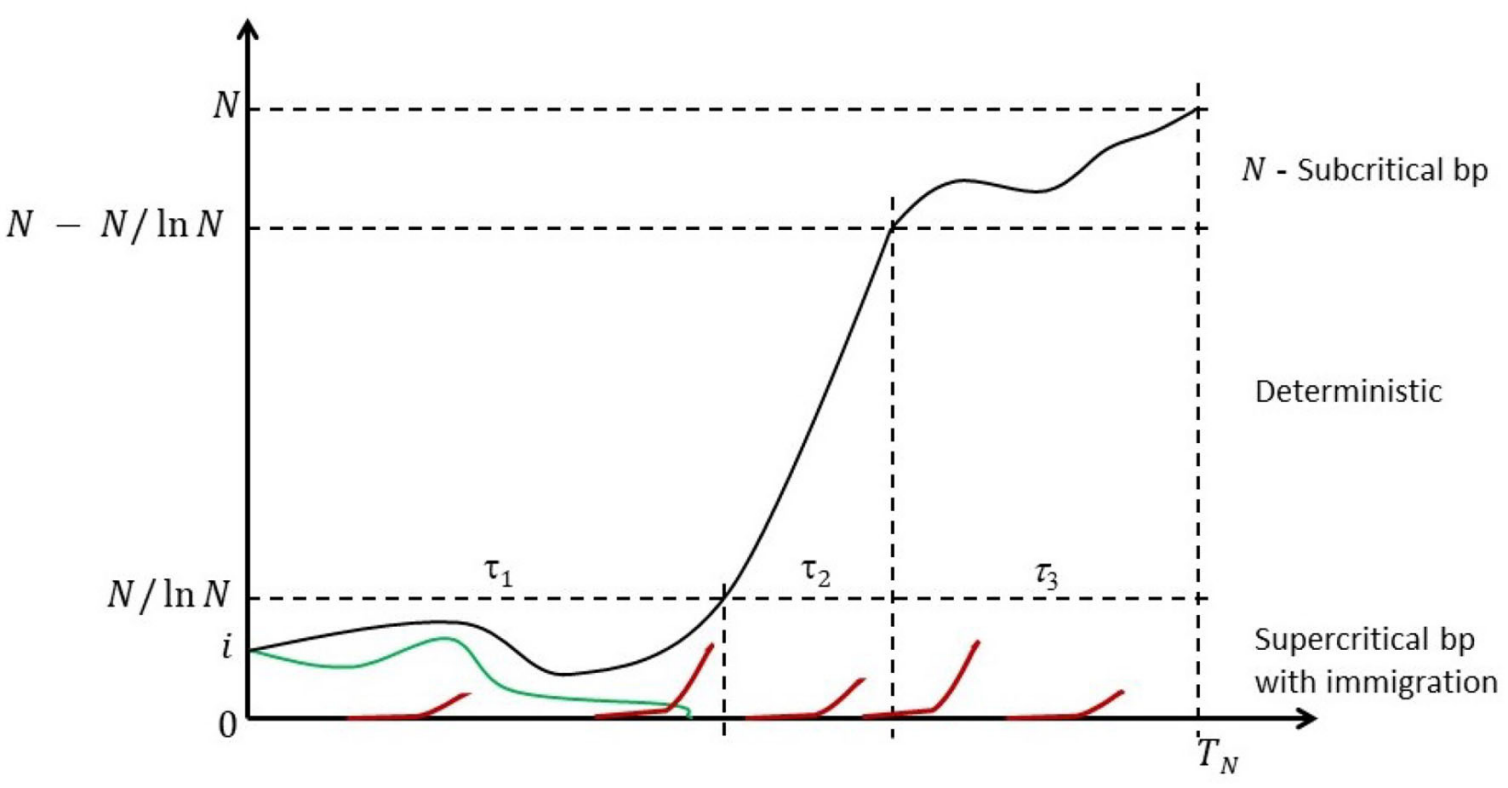
Anatomy of the Moran process with recurrent mutations. Some mutant count trajectories may become transiently extinct (green line), but they will be resurrected by one of the recurring mutations events (red lines). Eventually the mutants are fixed.

Because the state space is finite, the chain is eventually absorbed in the state *N* at random time *T*_*N*_; cf. Figure 1 for a heuristic illustration.

### 2.3. Simulation of Trajectories of the Moran Process With Recurrent Mutation

**Figure 2:**
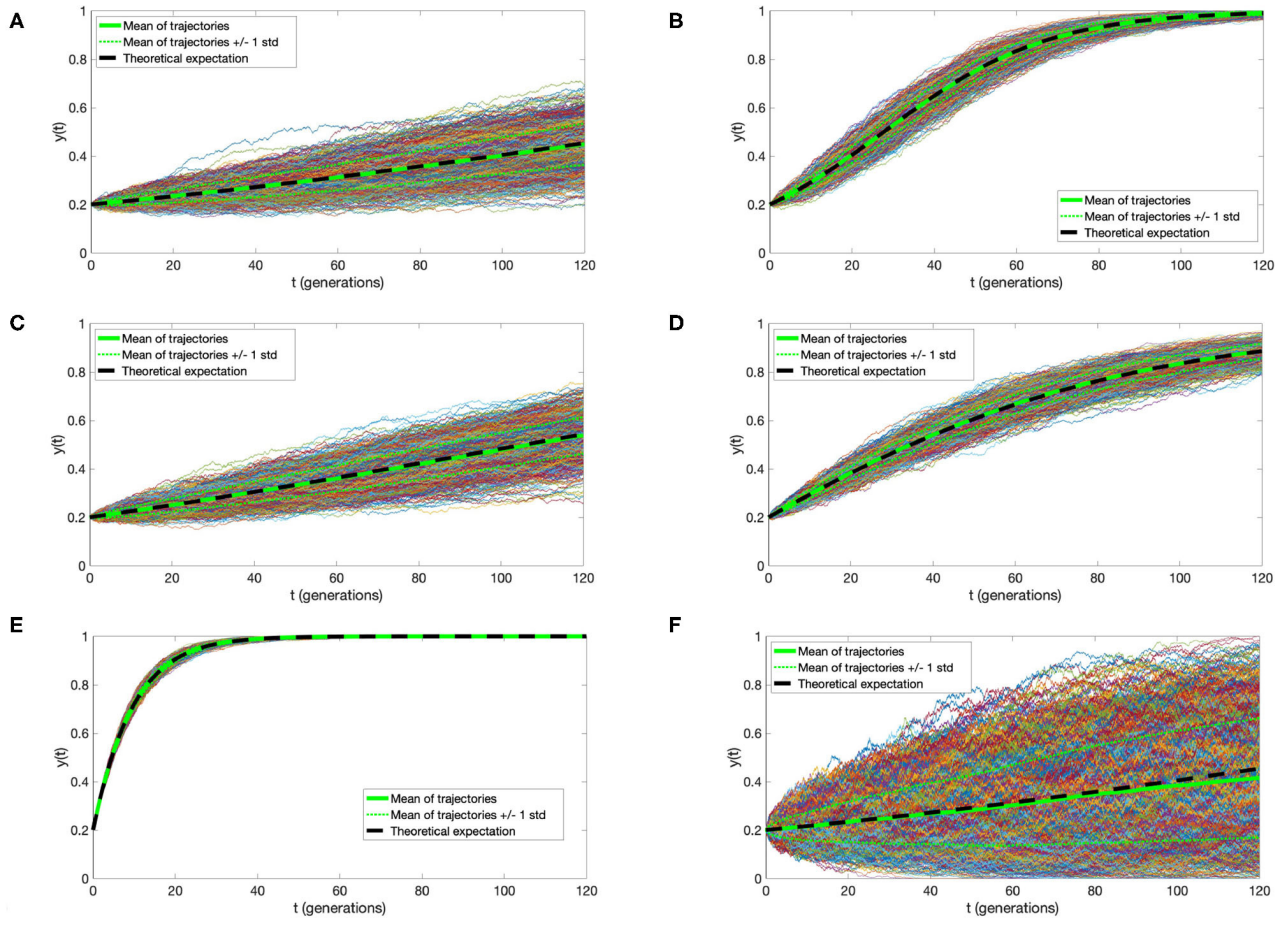
Trajectories of the Moran process with directional selection and recurrent mutation. One thousand simulations were plotted, with thicker green line denoting their mean, thinner green lines mean ± standard deviation, and black dashed line the solution of Equation (4). Parameter values **(A)** *s* = 0.01, μ = 0.000001, **(B)** *s* = 0.05, μ = 0.000001, **(C)** *s* = 0.01, μ = 0.001, **(D)** *s* = 0.01, μ = 0.01, **(E)** *s* = 0.01, μ = 0.1; and *N* = 10000, *t*_0_ = 0, *y*_0_ = 0.2 in **(A–E)**. **(F)** For comparison, trajectories under *s* = 0.01, μ = 0.1 as in **(A)**, but with *N* = 1, 000 are depicted, illustrating the impact of the cell count on variance of the process. In all panels, time is expressed in average cell generation units, μ in inverse time units, and *s* is dimensionless.

Simulation of a time-continuous Markov Chain is based directly on application of the transition intensities as expressed in Equation (1). Briefly, if the mutant cell chain *X*(*t*) is in state *i* at time *t*, then the time to the next jump is a random variable τ distributed exponentially with parameter *q*_*i,i*−1_ + *q*_*i, i*+1_. The direction of the jump at time *t* + τ is then decided by a random choice, *i*→*i* − 1 with probability $\frac{q_{i,i-1}}{q_{i,i-1}+q_{i,i+1}}$ and *i*→*i*+1 with probability $\frac{q_{i,i+1}}{q_{i,i-1}+q_{i,i+1}}$, respectively. This algorithm (know also popularly as the Gillespie algorithm) is based on the properties of holding times and jumps of time-continuous Markov Chain, as explained for example in the book (Grimmett and Stirzaker, 2001). Simulations depicted in Figure 2 were executed using this algorithm.

### 2.4. Approximation of the Moran Process With Recurrent Mutation and Estimation of Selection Coefficient and Mutation Rate

In the current study, we are not as much concerned with a mathematically rigorous theory of the Moran process with selection and recurrent mutation, as with obtaining computable expressions that lead to ballpark estimates of the selection coefficient and mutation rate. Based on transitions spelled out in Equation (1), we obtain the following expression for the conditional expectations:

(2) $$\begin{array}{r} \text{E}\left[X\left(t+\Delta t\right)|X\left(t\right)=x\right]=x+\left(\frac{\left(N-x\right)xs}{N}+\mu \left(N-x\right)\right)\Delta t+o\left(\Delta t\right) \end{array}$$

Corresponding expression for variance is more involved. From Equation (2), assuming that *x* = *X*(*t*) can be replaced by its expectation and denoting the latter by *x*(*t*) we formally obtain the following ordinary differential equation (ODE) for *x*(*t*).

(3) $$\begin{array}{r} ẋ\left(t\right)=\left(\frac{\left(N-x\left(t\right)\right)x\left(t\right)s}{N}+\mu \left(N-x\left(t\right)\right)\right),t\in \left[t_{0},t_{1}\right] \end{array}$$

Following a change of variables *y*(*t*) = *x*(*t*)/*N* ∈ [0, 1], this leads to

(4) $$\begin{array}{r} ẏ\left(t\right)=\left(1-y\left(t\right)\right)y\left(t\right)s+\mu \left(1-y\left(t\right)\right),t\in \left[t_{0},t_{1}\right] \end{array}$$

This latter equation has an explicit solution

(5) $$\begin{array}{r} y\left(t\right)=\frac{1-\left(\mu /s\right)\alpha_{0}\exp\left(-\left(\mu +s\right)\left(t-t_{0}\right)\right)}{1+\alpha_{0}\exp\left(-\left(\mu +s\right)\left(t-t_{0}\right)\right)},t\in \left[t_{0},t_{1}\right] \end{array}$$

where

(6) $$\begin{array}{r} \alpha_{i}=\left(1-y_{i}\right)/\left(y_{i}+u\right),y_{i}=y\left(t_{i}\right),i=0,1,\text{and}u=\mu /s \end{array}$$

This curve is very similar to that derived in the initial part of the well-known study by Gerrish and Lenski (1998), under branching process hypotheses. Let us also notice that population size *N* does not play a role in the expression for *y*(*t*). However, as evidenced by a comparison between the simulations in Figures 2A,F, larger *N* reduces process variance and the slight bias of *y*(*t*) as the estimate of *X*(*t*)/*N*.

Let us note that Equation (5) yields

(7) $$\begin{array}{r} \frac{1-y\left(t\right)}{y\left(t\right)+\mu /s}=\alpha_{0}\exp\left(-\left(\mu +s\right)\left(t-t_{0}\right)\right), \end{array}$$

which after substitution *t* = *t*_1_ yields

(8) $$\begin{array}{r} \alpha_{1}=\frac{1-y_{1}}{y_{1}+u}=\alpha_{0}\exp\left(-\left(\mu +s\right)\left(t_{1}-t_{0}\right)\right), \end{array}$$

which yields

(9) $$\begin{array}{r} \mu +s=\frac{\ln\left(\alpha_{1}/\alpha_{0}\right)}{t_{1}-t_{0}} \end{array}$$

The latter can be written alternatively as

(10) $$\begin{array}{r} s=\frac{1}{\left(1+u\right)}\frac{\ln\left(\alpha_{1}/\alpha_{0}\right)}{\left(t_{1}-t_{0}\right)} \end{array}$$

Knowing *y*_0_ and *y*_1_ (and therefore also knowing α_0_ and α_1_), we can thus now find the set of values (*s*, μ) such that *y*_*i*_ = *y*(*t*_*i*_) *i* = 0, 1.

The latter expression embodies the trade-off between selection and mutation. To understand it, let us notice that the RHS of Equation (10) is equal to $C=\frac{\ln\left(\alpha_{1}/\alpha_{0}\right)}{\left(t_{1}-t_{0}\right)}$ if *u* = 0, and it changes very little if *u* is small. The magnitude of *C* (which is the estimate of *s* when μ = 0) as computed from data varies between 0.002 and 0.05 if we disregard the sole negative value −0.059. To significantly influence (i.e., by say 10%) the lowest estimate 0.002, the mutation per cell per nucleotide rate should be equal to at least 0.0002, which is five orders of magnitude higher than the standard human rate. We use per nucleotide rates since we are discussing specific mutation sites in each case.

**Figure 3:**
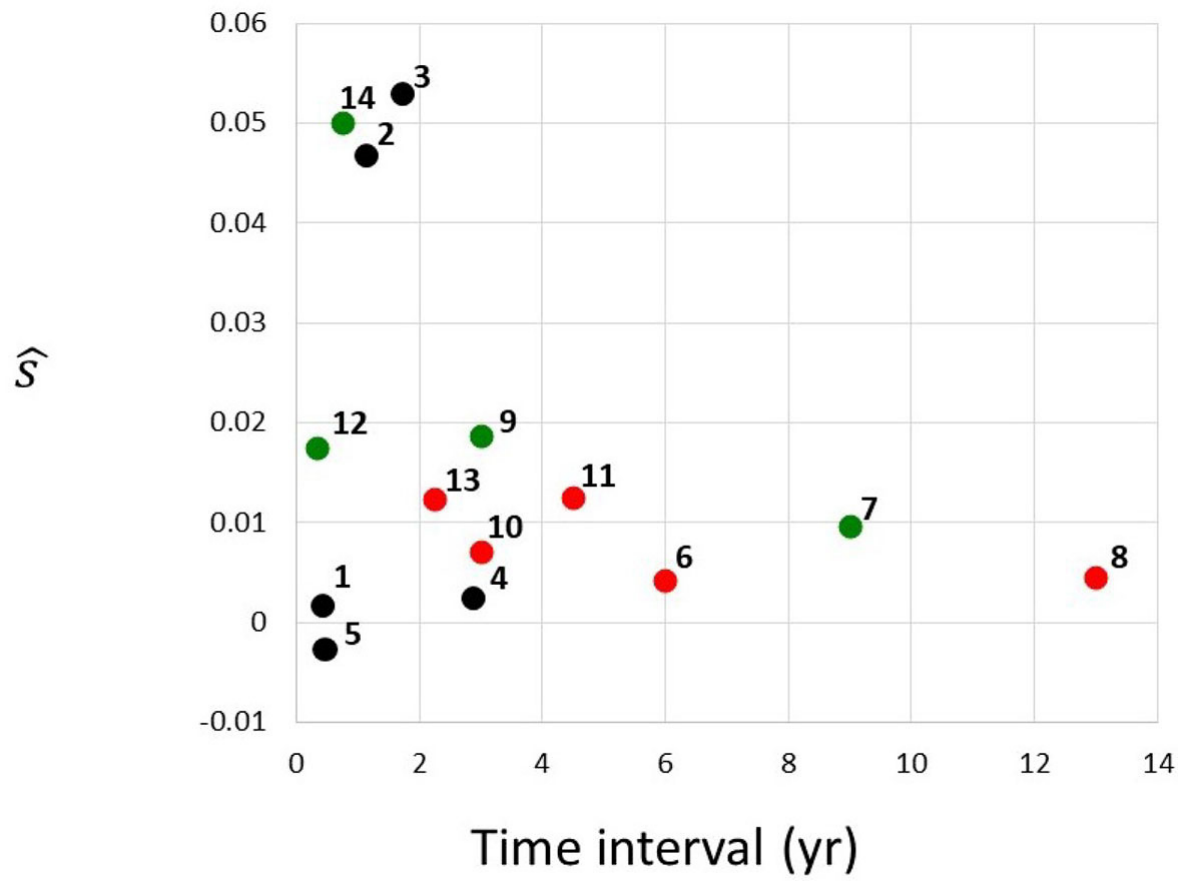
Estimated selection coefficients from Table 1, plotted against the time interval between the first and the second instance of sequencing. Red, MDS; green, AML; black, unclassified. Numbering of cases follows the identifiers in Table 1.

In summary, estimates depicted in Table 1 and Figure 3 were obtained using the method of the preceding paragraph under assumption μ = *u* = 0.

**Table 1:** Tabular summary of truncated receptor data from three publications, and resulting estimates of selection coefficient for the Moran model without recurrent mutation.

**Identifier**	**References**	**Figure**	**Case**	**Phase**	***y*_0_**	***y*_1_**	***t*_1_−*t*_0_**	**λ**	**α_1_**	**α_2_**	**ŝ**	**Mutation**
1	Klimiankou et al. (2019)	Figure 4E	CN pt. 21		0.13	0.14	0.45	24	0.15	0.16	0.002	Q749
2			CN pt. 11		0.03	0.10	1.15	24	0.03	0.12	0.047	Q754
3			CN pt. 27		0.01	0.06	1.75	24	0.01	0.06	0.053	Q741
4			CN pt. 19		0.13	0.15	2.90	24	0.15	0.18	0.002	Y752
5			CN pt. 13		0.13	0.07	0.45	24	0.14	0.08	-0.059	Q741
6	Beekman et al. (2012)	Figure S4	pt. ph. 1	MDS	0.06	0.11	6.00	24	0.07	0.12	0.004	D715
7			pt. ph. 2	AML	0.11	0.49	9.00	24	0.12	0.97	0.010	D715
8	Skokowa et al. (2014)	Figure S3	pt. 6 ph. 1	MDS	0.24	0.56	13.00	24	0.32	1.27	0.004	Q726X
9			pt. 6 ph. 2	AML	0.56	0.83	3.00	24	1.27	4.88	0.019	Q726X
10			pt. 10	MDS	0.01	0.02	3.00	24	0.01	0.02	0.007	Q726P
11			pt. 16 ph. 1	MDS	0.10	0.30	4.50	24	0.11	0.43	0.012	Q720X
12			pt. 16 ph. 2	AML	0.30	0.33	0.33	24	0.43	0.49	0.017	Q720X
13			pt. 19 ph. 1	MDS	0.28	0.43	2.25	24	0.39	0.75	0.012	Y729X
14			pt. 19 ph. 2	AML	0.43	0.65	0.75	24	0.75	1.86	0.050	Y729X

*Figure numbers are these in original publications. MDS/AML column based on the disease history of the patient in Beekman et al. (2012) and Figure 3 in the Supplement to Skokowa et al. (2014); Klimiankou et al. (2019) is listing “CN-MDS/AML” as a single category. y_0_, initial fraction of mutant receptor, y_1_, final fraction of mutant receptor, t_1_ − t_0_, duration of time interval (years), λ, average count of cell divisions (year^−1^), also equal to the inverse of the expected interdivision time (years), α_0_, α_1_, as defined in Equation (8), and ŝ, estimate of the selection coefficient. “pt.” is patient, “ph.” is phase*.

### 2.5. Empirical Observations of Increase in Mutant Receptor Frequency Over Time

Several papers documented the process of increase of the frequency of mutant receptor, based on genome sequencing of bone marrow cells of the SCN patients, in two or more time points. We focus our attention on three of these papers (Beekman et al., 2012; Skokowa et al., 2014; Klimiankou et al., 2019). In these papers, patient data were recorded with changing frequency of mutant receptors over time. Of these cases, we selected only the ones that displayed unambiguous monotonous trend. Theses case are listed in Table 1. The estimates that are obtained using expression (10) are ambiguous since the expression provides only a relationship between *s* and μ. However, if *s* estimated under the hypothesis that μ is small, which is plausible unless the mutation rate is orders of magnitude higher than normal, then the estimates of *s* differ only slightly from those obtained under μ = 0, as explained in the preceding subsection. This effect is very similar to that observed in simulations in Wojdyla et al. (2019).

## 3. Results

### 3.1. Approximate Mean Expression vs. Simulations

We address here the accuracy of the agreement of the approximate expression (5) for expected value of the Moran process with directional selection and recurrent mutations, with direct simulations. Figure 2, presents a comparison of 1,000 simulated trajectories and their mean and standard deviation to the *y*(*t*) function. We observe an almost complete agreement of the approximate mean and simulation average, which become indistinguishable with cell count *N* increasing from 1, 000 to 10, 000 [compare panels (A–F)]. Additionally, the simulation variance decreases almost inversely proportionally to *N*.

It is very important to compare the influence of selection coefficient *s* and mutation rate μ on the expectation of the process. If μ does not exceed 0.001 per generation, its influence can be disregarded, while the influence of *s* is decisive [panels (A–C)]. Only when μ reaches 0.01, its influence becomes important. In the estimates of the selection coefficient *s*, based on expression (10), this effect is represented by the coefficient *u* = μ/*s* (also present in expressions for α_0_ and α_1_), the magnitude of which determines the departure from the case μ = 0.

### 3.2. Estimates of Selection Coefficients

Table 1 depicts the estimates of the selection coefficient *s* obtained using Equation (10) with μ = 0. All details of the data used are included in the Table 1. The estimates depend on the assumed average interdivision time of the HSC (including some self-renewing CMP). It is assumed to be equal to 1/24 of 1 year (15 days). Changing this assumption leads to different estimates, as it can be tested by modifying the parameter λ in the spreadsheet (λ equals the inverse of the interdivision time). Overall, the estimates span a range from 0 to 0.05, with the exception of patient 13 of publication (Klimiankou et al., 2019) who has a negatively estimated selection coefficient.

Figure 3 depicts estimated selection coefficients ŝ from Table 1, plotted against the time interval *t*_1_ − *t*_0_ between the first and the second instance of sequencing. There seems to exist a negative association between ŝ and *t*_1_ − *t*_0_. In addition, the MDS cases (red circles), seem to have lower values of ŝ than the AML cases (green circles). Cases labeled as “CN-MDS/AML” in Klimiankou et al. (2019) are denoted by black circles.

## 4. Discussion

The results of the present paper provide estimates of the selection coefficients that may underlie the fixation of the mutant G-CSF receptor in the SCN to MDS transition, which are consistent with the range deduced in Wojdyla et al. (2019) based on epidemiological evidence. Let us emphasize that our initial and final fractions of mutant receptor data come from sequencing of samples from patients with SCN. Availability of these sequencing data in papers (Beekman et al., 2012; Skokowa et al., 2014; Klimiankou et al., 2019) is at the stem of our results.

Severe congenital neutropenia is not the only inherited BMF syndrome with predisposition to MDS and AML; however, we believe that SCN provides the most robust and accurate disease to model because acquisition of *CSF3R* mutation is so common (70-80%) as a secondary hit (see discussion of sources in Wojdyla et al., 2019). On the other hand, *TP53* mutations in Shwachman-Diamond are controversial in that the mutations do not augur for transformation (Xia et al., 2018). Furthermore, the prevalence of mutations in *TP53* and in other genes such *RUNX1* in Fanconi anemia or dyskeratosis congenita appears to be much less than that of *CSF3R* in SCN (Chao et al., 2017; Lane, 2017; Kirschner et al., 2018). Despite this, modified Moran model might be applicable to other bone marrow failure syndromes that are associated with leukemia transformation. Since the variant allele frequencies of *CSF3R* is not reported for most of these rare patients, but our model provides an accurate prediction, it is conceivable that uncommon secondary mutations, such as *TP53* or *PPM1D* or *RUNX1*, could be used in our modified Moran model.

We do not use the cases with non-monotonic change in variant receptor frequency. The reason is that, at this range of frequency, Moran model is very unlikely to exhibit persistent reversals. Therefore, it is more likely that factors that cannot be included in the Moran model play a role.

One of the important questions in understanding cancer evolution is the balance among different genetic forces, such as mutation and selection. The problem has been studied for solid cancers, e.g., by Ling et al. (2015). In essence, mutant frequency in the cell population can increase in a similar way with different (negatively associated) values of *s* and μ. In particular, if a fit to the mutant frequency increase observed over a time interval is obtained under μ = 0, as in Table 1, then under μ > 0, the estimate of *s* will only be smaller. The decrease will depend on the value of *u* = μ/*s*. However, as discussed in the Results, unless the mutation rate in cells is five orders of magnitude higher than in normal cells, i.e., μ ≈ 10^−4^ per cell generation per nucleotide, mutation does not make much difference for the estimates. Therefore, recurrent mutation is an important factor only if the *CSF3R* mutation sites are extremely strong mutational hot-spots. We also examined the magnitude of the correlation coefficient, depending on whether the observed transition was from SCN to MDA or to AML (Figure 3), wherever the data have been available. The selection coefficients in the transitions to AML seem to be greater.

An additional effect may be due to the fact that not one, but several types of mutant receptors are observed in MDS. The most frequent is the truncated D715 variant, but there are a number of other, as documented in Beekman et al. (2012), Skokowa et al. (2014), and Klimiankou et al. (2019). Therefore, the basic mutation rate should be multiplied by the number of alternative mutants. Assuming that there are no more than 10 of these mutants, the effect does not seem to play a major role.

It is interesting to observe the apparent effect of ascertainment bias on the data from papers (Beekman et al., 2012; Skokowa et al., 2014; Klimiankou et al., 2019) which we use in our study. Figure 3 in the Results depicts estimated selection coefficients ŝ from Table 1, plotted against the time interval Δ*t* = *t*_1_ − *t*_0_ between the first and the second instance of sequencing. The negative association seen in Figure 3 may be explained by the fact that the second time at which the frequency of the mutant receptor is observed, arrives sooner if the progression of the disease is faster, i.e., when the coefficient *s* is higher. This trend may also lead to our estimated *s* being in general an overestimate, under the assumption that cases with very low *s* are never diagnosed. Impact of ascertainment bias on estimates of progression in solid cancers has been studied (see, e.g., Kimmel and Flehinger, 1991), and similar methods may be used. However, such study exceeds the framework of the current paper.

## Data Availability Statement

All datasets generated for this study are included in the article.

## Author Contributions

All authors listed have made a substantial, direct and intellectual contribution to the work, and approved it for publication. Specifically, MK conceived the study and derived mathematical expressions, SC searched for relevant data and interpreted the model in the context of data, and KD designed and carried out Moran model computations and simulations.

## Conflict of Interest

The authors declare that the research was conducted in the absence of any commercial or financial relationships that could be construed as a potential conflict of interest.
